# Cohort profile: the Kyrgyzstan InterSectional Stigma (KISS) injection drug use cohort study

**DOI:** 10.1186/s12954-022-00633-5

**Published:** 2022-05-25

**Authors:** Laramie R. Smith, Natalia Shumskaia, Ainura Kurmanalieva, Thomas L. Patterson, Dan Werb, Anna Blyum, Angel B. Algarin, Samantha Yeager, Javier Cepeda

**Affiliations:** 1 Division of Infectious Diseases and Global Public Health, Department of Medicine, University of California San Diego, 9500 Gilman Drive, Mail Code 0507, La Jolla, CA 92093-0507 USA; 2 AIDS Foundation–East West in the Kyrgyz Republic, Bishkek, Kyrgyzstan; 3 Department of Psychiatry, University of California, San Diego, La Jolla, CA USA; 4 Centre On Drug Policy Evaluation, St. Michael’s Hospital, Toronto, Canada; 5 Department of Epidemiology, Johns Hopkins Bloomberg School of Public Health, Baltimore, MD USA

**Keywords:** People who inject drugs, HIV, Stigma, Eastern Europe and Central Asia

## Abstract

**Background:**

In Kyrgyzstan and other Eastern European and Central Asian countries, injection drug use and HIV-related intersectional stigma undermines HIV prevention efforts, fueling a rapidly expanding HIV epidemic. The Kyrgyzstan InterSectional Stigma (KISS) Injection Drug Use Cohort is the first study designed to assess the impact of drug use, methadone maintenance treatment (MMT) and HIV stigma experiences among people who inject drugs (PWID) on HIV prevention service utilization.

**Methods:**

Adult PWID were recruited from Bishkek city and the surrounding rural Chuy Oblast region in northern Kyrgyzstan via modified time location sampling and snowball sampling. All participants completed a baseline rapid HIV test and interviewer-administered survey. A subsample of participants were prospectively followed for three months and surveyed to establish retention rates for future work in the region. Internal reliability of three parallel stigma measures (drug use, MMT, HIV) was evaluated. Descriptive statistics characterize baseline experiences across these three stigma types and HIV prevention service utilization, and assess differences in these experiences by urbanicity.

**Results:**

The KISS cohort (*N* = 279, 50.5% Bishkek, 49.5% Chuy Oblast) was mostly male (75.3%), ethnically Russian (53.8%), median age was 40 years old (IQR 35–46). Of the 204 eligible participants, 84.9% were surveyed at month 3. At baseline, 23.6% had a seropositive rapid HIV test. HIV prevention service utilization did not differ by urbanicity. Overall, we found 65.9% ever utilized syringe service programs in the past 6 months, 8.2% were utilizing MMT, and 60.8% met HIV testing guidelines. No participants reported PrEP use, but 18.5% had heard of PrEP. On average participants reported moderate levels of drug use (mean [*M*] = 3.25; *α* = 0.80), MMT (*M* = 3.24; *α* = 0.80), and HIV stigma (*M* = 2.94; *α* = 0.80). Anticipated drug use stigma from healthcare workers and internalized drug use stigma were significantly higher among PWID from Bishkek (*p* < 0.05), while internalized HIV stigma among PWID living with HIV was significantly greater among PWID from Chuy Oblast (*p* = 0.03).

**Conclusion:**

The KISS cohort documents moderate levels of HIV-related intersectional stigma and suboptimal engagement in HIV prevention services among PWID in Kyrgyzstan. Future work will aim identify priority stigma reduction intervention targets to optimize HIV prevention efforts in the region.

**Supplementary Information:**

The online version contains supplementary material available at 10.1186/s12954-022-00633-5.

## Background

The HIV epidemic in Eastern Europe and Central Asia (EECA) is rapidly expanding despite reductions in HIV incidence achieved in other regions [1]. Most new HIV infections in EECA are occurring in former Soviet Republics, including Kyrgyzstan, situated along Afghan heroin trafficking routes destined to Russia [2]. People who inject drugs (PWID) comprise 51% of all people living with HIV in Kyrgyzstan, a mostly rural country of 6 million people [3]. HIV prevalence among PWID is approximately 14% in Kyrgyzstan, 70 times higher than in the general population (0.2%) and 45 times higher than in rural regions (0.31%) [4]. Of concern, HIV incidence is increasing in rural settings [5], while the lack of decentralized HIV prevention services for PWID in rural areas remains a key barrier to progress [6].

The Kyrgyz government is the regional vanguard of HIV prevention for PWID, implementing antiretroviral therapy (ART), syringe service programs (SSP) and methadone maintenance treatment (MMT) services within community and prison settings [7, 8]. Yet, success is limited by suboptimal availability and low uptake of these critical services with only 4% of PWID engaged in MMT [4], and among those living with HIV, less than one-third are on ART [4]. Low engagement of PWID in these preventive services is a concern and is believed to be the primary driver of growing HIV incidence among heterosexual (non-injecting) partners, bridging the epidemic from PWID to the general population [9].

Stigma remains a pervasive barrier to HIV prevention efforts across EECA [10, 11]. In Kyrgyzstan, PWID cited stigma and discrimination, including fear of police encounters, as a primary barrier to SSP use [12]. PWID reported avoiding HIV testing due to fears of having ones’ HIV status disclosed if they tested HIV-positive, while discriminatory treatment from service providers and fear of having one’s drug use status disclosed by staff or if one was seen within the vicinity of drug treatment centers was a barrier to accessing drug treatment [6, 10]. A study with recently incarcerated PWID in the EECA observed that MMT was viewed by PWID in Kyrgyzstan as a ‘treatment of last resort’ for PWID that are very sick [13]. Such findings suggest that MMT use may confer its own stigma and amplify experiences of drug use stigma among PWID. Further, disparities in ability to access basic HIV prevention services exist between urban and rural areas in Kyrgyzstan and other EECA countries, which may lead to greater anticipated stigma in rural areas where anonymity is more limited [6, 10].

First operationalized by Michele Tracey Berger [14], intersectional HIV-related stigma research is grounded in intersectionality, an analytical lens originating from Black feminist theory [15, 16]. Applied to PWID in the EECA region, this approach underscores the importance of examining multilevel experiences of interlocking status-based oppression related to drug use, MMT, and HIV, as well as contexts, such as urbanicity among others, that may further influence how these stigmas are experienced. To date, stigma research with PWID has been largely unidimensional, examining drug use, MMT, and HIV stigma in the region independent of one another [17–21]. To our knowledge, two studies have examined the intersection of drug use and HIV stigma in the EECA region with mixed findings. Among PWID living with HIV in Russia, Calabrese and colleagues (2016) observed high levels of internalized drug use and HIV stigmas were associated with poorer physical health and HIV care utilization, whereas Vetrova and colleagues (2021) observed only high levels of drug use stigma was associated with poor HIV care access [22, 23].

Qualitative stigma research in the region [9, 24, 25], as well as our own quantitative stigma research in the United States further suggest that different sources of stigma may uniquely impact health behaviors and outcomes [26–28]. For example, healthcare workers may hold more stigmatizing beliefs toward drug use impeding healthcare access [29], while family members may be more likely to view MMT as replacing one drug for another restricting access to economic and social support that could facilitate sustained engagement in MMT [30, 31]. Likewise, anticipated HIV stigma may be greater among PWID given elevated risk of direct transmission through injection-related interactions reducing motivation to know one’s HIV status [32].

To address these gaps, the Kyrgyzstan InterSectional Stigma (KISS) Injection Drug Use Cohort study aims to assess the impact of HIV-related intersecting stigmas among PWID on HIV prevention service utilization using parallel stigma measures designed to capture multilevel stigma mechanisms and distinguish between key stigma sources. This paper aims to describe the study methods, measures, and baseline characteristics of the KISS Cohort, and assess differences in HIV-related intersectional stigma by urbanicity and key stigma sources.

## Methods

### Study aims

The primary aim of the KISS cohort study is to systematically assess and characterize potentially intersecting experiences of drug use, MMT, and HIV stigma among a diverse sample of urban and rural Kyrgyz PWID to identify optimal HIV prevention intervention strategies in subsequent analyses.

#### Hypothesis 1

Total drug use, MMT, and HIV stigma experiences will be significantly higher among rural PWID compared to urban PWID.

#### Hypothesis 2

Compared to other stigma sources across drug use, MMT, and HIV stigma types at baseline, (a) anticipated drug use stigma will be highest from healthcare workers, (b) anticipated MMT stigma will be highest from family members, and (c) anticipated HIV stigma would be highest from other PWID.

The secondary aim of the KISS cohort study was to establish and refine retention protocols and obtain retention estimates to inform larger scale observational cohort and intervention research in the region.

### Study setting and population

**Fig. 1:**
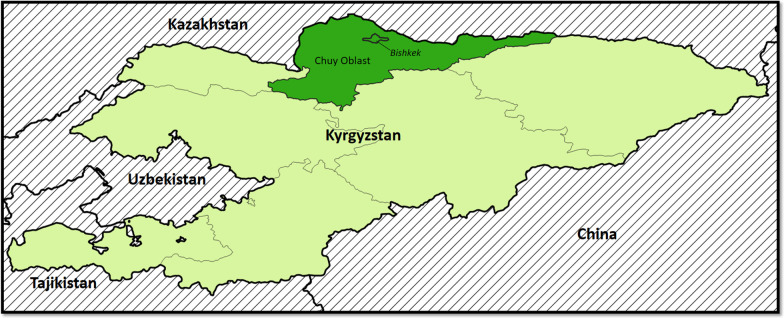
Map of Bishkek City and surrounding Chuy Oblast recruitment regions in Northern Kyrgyzstan

We conducted the KISS cohort study in the capital city of Bishkek and the surrounding rural province of Chuy Oblast in northern Kyrgyzstan (Fig. 1). We selected these two study sites because they are home to the highest proportion of PWID and new HIV diagnoses in Kyrgyzstan [4], and because these sites are reflective of both urban and rural settings, where HIV incidence continues to rise.

Eligibility criteria for all participants were: 1) 18 years of age or older, 2) injection drug use behavior in the past 30 days with visual confirmation of injection marks, 3) agree to a rapid HIV test, and 4) residing in either Bishkek or Chuy Oblast with no plans to relocate in the next 12 months. In addition, participants recruited via snowball sampling was restricted to PWID not actively engaged in SSP or MMT services.

### Study sampling and recruitment

**Fig. 2:**
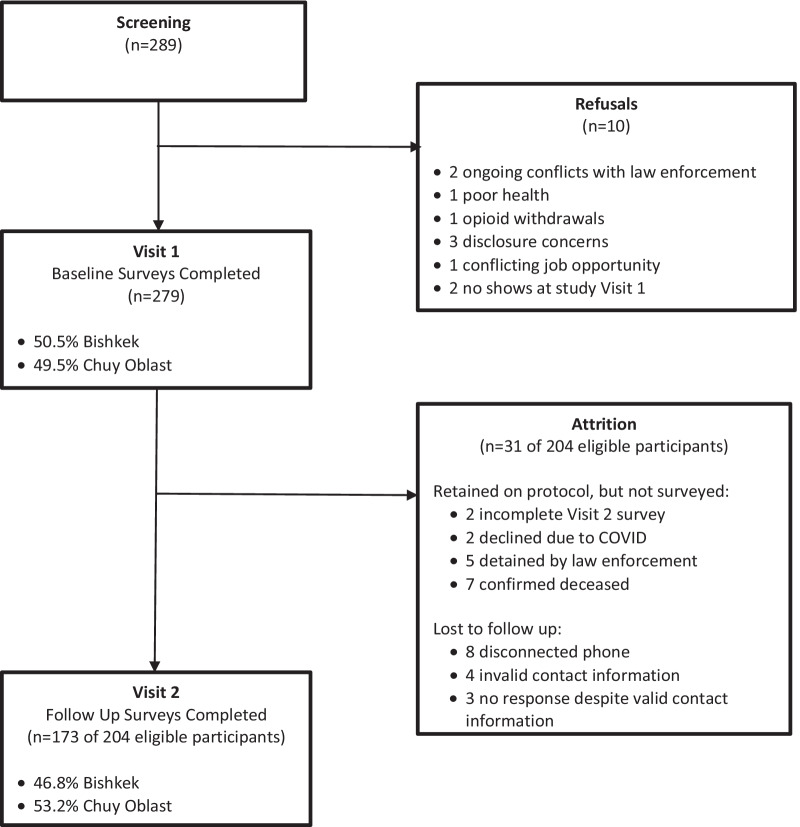
Kyrgyzstan InterSectional Stigma (KISS) injection drug use cohort flow chart

To ensure we reached a diverse sample of PWID, we applied two non-random purposive sampling frameworks (see Fig. 2). Our sample size target was 279 PWID with at least 50% of the sample residing in Chuy Oblast to be adequately powered to assess small effects in the total sample (*σ* = 0.23), and medium effects by urbanicity (*σ* = 0.31) in future analyses using latent variable structural equation modeling. We powered for these analyses to simultaneously test the relative contributions of HIV-related intersectional stigmas (HIV, drug use, MMT) and mechanisms (structural, interpersonal, individual); to identify which associations should be prioritized as intervention targets to improve engagement in HIV prevention services [33].

In response to COVID19 related restrictions limiting public social gatherings, we employed a modified venue-based time location sampling approach April 5, 2021 to May 6, 2021. Outreach workers of a local harm reduction agency partnered with study investigators to coordinate recruitment efforts. Study investigators randomly assigned 15 outreach workers in Bishkek and 17 outreach workers in Chuy Oblast different days of the week (time) to recruit from a specific neighborhood (venues) where they served PWID in Bishkek (15 neighborhoods) and Chuy Oblast (20 neighborhoods). Study investigators randomly assigned additional enrollment targets to each outreach worker with the aim of enrolling ≥ 50% PWID age 20–39 (among whom 67% of new HIV diagnoses occur [34], ≥ 20% female PWID, and ≥ 33% PWID living with HIV. Outreach workers reported the numbers and reasons for refusals to participate they encountered during their specified time location sampling block. In addition, we employed snowball sampling to enroll harder-to-reach PWID less engaged in HIV prevention services. From October 6, 2021 to October 28, 2021, study investigators invited PWID enrolled in the study and all outreach workers to refer other PWID they knew who reported they were not actively engaged in SSP or MMT services. Given the nature of this method, refusal data were not systematically collected. All respondents who contacted study staff were eligible and agreed to participate.

### Study procedures

Trained community-connected research assistants informed potential participants about study procedures and screened them for eligibility. Research assistants consented eligible PWID, completed a standard study locator form, and provided participants with a day and time to complete their baseline study visit (Visit 1). Study visits were conducted at a local harm reduction agency. We structured study visits to maximize COVID social distancing guidelines within the agency.

At Visit 1, participants initially underwent rapid HIV testing and then met with a trained interviewer to complete the baseline survey. Following the survey, participants received their HIV test results and posttest counseling. Certified HIV testers used OraQuick™ Rapid HIV antibody test to conduct all rapid HIV tests. Previously undiagnosed PWID were linked to the Republican AIDS Center in Bishkek for confirmatory testing per national protocol.

Visit 2 surveys were scheduled 3 months post-baseline for the 204 participants recruited via modified venue-based time location sampling due to funding and time restrictions. The goal of Visit 2 was to establish retention estimates for future observational and intervention research in the region. Using participant locator data, staff systematically contacted participants to schedule Visit 2 and document reasons participants could not be located or could not complete the follow-up survey. Participants were classified as retained on protocol if the participant completed the survey, staff spoke with the participant, or spoke with a contact provided by the participant at baseline who confirmed the participant was detained by police or confirmed the participant had passed. Cause of death was documented as reported by the participants’ contact. We classified participants not retained on protocol as lost to follow-up.

Baseline and follow-up surveys lasted approximately 45–60 min each. Both surveys captured the same questions described below, with the following exceptions. The follow-up survey excluded lifetime survey items asked at baseline and we changed all past 6 months reference periods to the past 3 months at follow-up. The follow-up survey also included brief measures assessing experiences of anxiety, depression, and resilience (data not shown). In response to field observations, on July 6, 2021 we added cross-sectional items to assess the following four domains to all follow-up surveys and to snowball sample baseline surveys. These items assessed (i) bath salt use, (ii) most recent MMT dose and duration (data not shown), (iii) initiation of diphenhydramine use in relation to participants’ MMT history (data not shown), and (iv) the acceptability of accessing HIV prevention services via alternative service delivery models (data not shown). Each survey question had response options that included ‘don’t know’ or ‘prefer not to answer.’ An answer was required for each question to advance to the next page. Trained interviewers administered the surveys in Russian and entered responses directly into Redcap through a secure survey link. Participants were compensated 300 mobile phone units (~ $2.50 USD) for each study visit completed. We obtained ethics approval for all study activities from the University of California, San Diego Institutional Review Board (IRB) and GLObal Research Institute (GLORI) Foundation IRB, Kyrgyzstan.

### Baseline measures and variables of interest

We coded all ‘don’t know’ and ‘prefer not to answer’ responses as missing. All lifetime and past 6 months measures were coded (0 = No, 1 = Yes), unless otherwise noted. Item-specific response categories are indented in Tables 1 and 2.

**Table 1:** Baseline characteristics of study participants overall and by urbanicity (*N* = 279)

	*N* with response	Total *N* = 279	Bishkek *N* = *141*	Chuy Oblast *N* = *138*	*p*-value
	*n*	*n* (%)	*n* (%)	*n* (%)	
*Sociodemographics*					
Median age (IQR)	279	40 (35–46)	39 (35–44)	40 (34–47)	.340^a^
Gender	279				.283
Men		210 (75.3%)	110 (78.0%)	100 (72.5%)	
Women		69 (24.7%)	31 (22.0%)	38 (27.5%)	
Ethnicity	279				.096
Kyrgyz		44 (15.8%)	23 (19.9%)	16 (11.6%)	
Russian		150 (53.8%)	76 (53.9%)	74 (53.6%)	
Another ethnicity		85 (30.5%)	37 (26.2%)	48 (34.8%)	
Education	279				.260
< High school		99 (35.5%)	44 (31.2%)	55 (39.9%)	
High school		106 (38.0%)	55 (39.0%)	51 (37.0%)	
> High school		74 (26.5%)	42 (29.8%)	32 (23.2%)	
Any unstable housing past 6 months	279	124 (44.4%)	73 (51.8%)	51 (37.0%)	**.013**
HIV status	279				**.002**
HIV-negative		213 (76.3%)	95 (67.4%)	118 (85.5%)	
Known HIV-positive		61 (21.9%)	42 (29.8%)	19 (13.8%)	
Newly diagnosed HIV-positive		5 (1.8%)	4 (2.8%)	1 (0.7%)	
*Substance use characteristics*					
Median age at first injection (IQR)	279	20 (17–24)	20 (17–25)	20 (17–23)	.550^a^
Injection frequency past 6 months	279				.544^a^
Daily		23 (10.0%)	14 (9.9%)	14 (10.1%)	
More than once a week		76 (27.2%)	38 (27.0%)	38 (27.5%)	
Once a week		64 (22.9%)	29 (20.6%)	35 (25.4%)	
1–3 times a month		111 (39.8%)	60 (42.6%)	51 (37.0%)	
Drugs injected past 6 months					
Heroin	266	240 (90.2%)	124 (93.2%)	116 (87.2%)	1.00
Diphenhydramine	234	142 (60.7%)	70 (60.3%)	72 (61.0%)	.485
Stimulants	205	15 (7.3%)	10 (9.3%)	5 (5.2%)	.635
Benzodiazepines	204	24 (11.8%)	18 (12.8%)	6 (6.1%)	**.019**^b^
Bath salts^**c**^	181	39 (21.5%)	15 (17.0%)	24 (25.8%)	.196
Poly-injection drug use past 6 months	279				.957^a^
Injected 1 drug type		98 (35.1%)	50 (35.5%)	48 (34.8%)	
Injected 2 drug types		108 (38.7%)	53 (37.6%)	55 (39.9%)	
Injected ≥ 3 drug types		73 (26.2%)	38 (27.0%)	35 (25.4%)	
Hazardous drinking	277	94 (33.9%)	49 (34.8%)	45 (33.1%)	.770

^a^Mann–Whitney U test.

^b^Fishers exact test.

^c^Baseline salt use assessed among 248 participants surveyed after July 6, 2021. Statistically significant differences are in bold

**Table 2:** Baseline HIV risk and prevention behaviors of study participants overall and by urbanity (*N* = 279)

	*N* with response	Total *N* = 279	Bishkek *N* = *141*	Chuy Oblast *N* = *138*	*p*-value
	*n*	*n* (%)	*n* (%)	*n* (%)	
*HIV transmission risk behaviors*					
Injection risk past 6 months (*Y*/*N*)					
Inject in public	276	99 (35.9%)	53 (38.1%)	46 (33.6%)	.430
Prepared drug with unsafe water source	279	37 (13.3%)	17 (12.1%)	20 (14.5%)	.549
Shared injection equipment	279	46 (16.5%)	24 (17.0%)	22 (15.9%)	.808
Reused cooker or filter	276	95 (34.4%)	51 (36.7%)	44 (32.1%)	.424
Frontload/backload syringe with others	278	28 (10.1%)	18 (12.9%)	10 (7.2%)	.120
Injected with a used needle	278	27 (9.7%)	16 (11.4$)	11 (8.0%)	.330
Had drugs to inject but no sterile syringe	279	89 (31.9%)	45 (31.9%)	44 (31.9%)	.996
Accidental overdose	279				
Lifetime		188 (67.4%)	93 (66.0%)	95 (68.8%)	.608
Past 6 months		28 (10.0%)	15 (10.6%)	13 (9.4%)	.735
Sexual behaviors past 6 months					
Any vaginal or anal sex	279	218 (78.1%)	108 (76.6%)	110 (79.7%)	.529
Any condomless sex	218	149 (68.3%)	73 (67.6%)	76 (69.1%)	.812
Multiple partners (≥ 1)	218	154 (70.6%)	80 (74.1%)	74 (67.3%)	.270
Partner is HIV-seropositive	193	17 (8.8%)	12 (12.2%)	5 (5.3%)	.087
Partner is PWID	216	60 (27.8%)	29 (27.1%)	31 (28.4%)	.826
Male PWID with male partner	218	3 (1.4%)	2 (1.9%)	1 (0.9%)	.620^b^
Engaged in sex work	210	17 (8.1%)	14 (13.9%)	3 (2.8%)	**.003**
*HIV prevention service utilization*					
SSP use past 6 months	279				.308^a^
Never accessed SSP		95 (34.1%)	48 (34.0%)	47 (34.1%)	
Accessed SSP < once a month		10 (3.6%)	8 (5.7%)	2 (1.4%)	
Accessed SSP 1–3 times a month		81 (29.0%)	28 (19.9%)	53 (38.4%)	
Accessed SSP once a week		82 (29.4%)	52 (36.9%)	30 (21.7%)	
Accessed SSP > once a week		11 (3.9%)	5 (3.5%)	6 (4.3%)	
Methadone maintenance treatment (MMT)	279				
Currently on MMT		23 (8.2%)	11 (7.8%)	12 (8.7%)	.786
Previously used MMT		94 (33.7%)	50 (35.5%)	44 (31.9%)	.527
Never used MMT		162 (58.1%)	80 (56.7%)	82 (59.4%)	.650
HIV-negative/newly diagnosed serostatus (*n* = 218)					
HIV testing past 12 months	218	133 (61.0%)	60 (60.6%)	73 (61.3%)	.777
Heard of PrEP	218	41 (18.8%)	18 (18.2%)	23 (19.3%)	.771
Known HIV-positive serostatus (*n* = 57)					
Had ≥ 1 HIV care visit past 6 months	57	53 (93.0%)	37 (94.9%)	16 (88.9%)	.584^b^
Currently taking ART Adherence		56 (98.2%)	38 (97.4%)	18 (100.0%)	1.00^b^
Past 30 days ART adherence	57				
≥ 80% ART adherence		52 (91.2%)	35 (89.7%)	17 (94.4%)	1.00^b^
≥ 95% ART adherence		42 (73.7%)	26 (66.7%)	16 (88.9%)	.109^b^
100% ART adherence		22 (38.6%)	15 (38.5%)	7 (38.9%)	.975

Statistically significant differences are in **bold**.

^a^Mann–Whitney U test

^b^Fishers exact test

#### Sociodemographic measures

Urbanicity (residing in Bishkek or Chuy Oblast), baseline HIV status, age (continuous), gender identity, ethnicity, and education. We defined unstable housing as sleeping any place other than a house or apartment participants or someone they knew (i.e., partner, friend, relative) owned or rented in the past six months (e.g., sleeping in a hotel, shooting gallery, hospital, street, jail, boarding house, government shelter).

#### Substance use measures

Participants’ reported age at first injection (in years), how often they typically injected drugs in the past 6 months, and drug types injected in the past 6 months. Poly-drug use reflects the number of drug types injected in the past 6 months. This includes injection of diphenhydramine an antihistamine akin to Benadryl frequently used in the region [35]. Notably, salt use (i.e., bath salts) was added to the baseline and follow-up surveys beginning July 6, 2021 following observations by outreach workers that more clients were reporting salt use. Thus, ‘baseline’ salt use is only available for participants who were recruited via time location sampling that attended Visit 2 and all participants recruited via snowball sampling. Lifetime and past 6 months overdose (defined as having lost consciousness or stopped breathing as a result of taking narcotics by injection or any other route of administration). We summed responses to the AUDIT (10 items; range: 0–40) to classify participants as having hazardous (score 8–40) or non-hazardous drinking (score 0–8) using standardized cutoffs [36].

#### HIV transmission risk measures

Past 6 months engagement in high-risk injection behaviors (e.g., sharing injection equipment, reusing a cooker or filter) and injecting in context associated with higher HIV transmission (i.e., injecting in public spaces, having drugs to inject but did not knowing where to get a sterile syringes). Past 6 months sexual HIV transmission risk was computed among participants who engaged in any anal or vaginal sex to classify high-risk sexual behaviors (i.e., condomless sex, multiple partners) and partner types that may increase potential exposure to HIV (e.g., partners who inject drugs). Engaging in sex work reflects participants who indicated that they earned income via sex work or reported engaging in sex in exchange for money, drugs, or other goods in the past 6 months.

#### HIV prevention service utilization measures

Frequency of accessing SSP services in the past six months, history of engaging in MMT (currently on MMT, previously used MMT, never used MMT). Among participants who were HIV-seronegative (or newly diagnosed) at baseline, we assessed whether they met local HIV testing guidelines (i.e., ≥ 1 HIV test every 12 months) by calculating time since the month and year of their last HIV test prior to baseline. We coded participants who had never previously tested as having not tested in the previous 12 months. Participants also indicated whether they had ever heard of or taken pre-exposure prophylaxis (PrEP) which became available in Kyrgyzstan in 2020. Participants who were known to be HIV-seropositive at baseline self-reported the number of HIV care appointments attended in the past 6 months, if they were currently taking ART, and what proportion of their ART they took as prescribed in the past 30 days on a single visual analogue scale (range: 0–100%) [37].

#### HIV-related intersectional stigma measures

We used three parallel drug use, MMT, and HIV stigma measures (18 items each) first validated in the United States. Aligned with work by Earnshaw (2009) and Gamarel (2018), stigma items were framed to capture stigma toward current drug use, or expectations of future rejection/self-devaluation whether one were to test HIV-positive or initiate MMT [38–40]. Informed by local qualitative stigma research with PWID [17, 25, 41, 42], three items were developed for this study to capture anticipated consequences of structural stigma (i.e., fear of police interactions, fear of being registered in government systems, fear of being denied housing or employment). Stigma sources most likely to influence HIV prevention behaviors among PWID (i.e., anticipated discrimination from family, healthcare workers, and other PWID) were assessed drawing from previously validated interpersonal stigma measures developed to capture anticipated stigma from three distinct stigma sources [26]. Previously validated individual-level stigma items assessed experiences of internalized drug use stigma, as well as stereotyped beliefs and prejudicial attitudes toward people on MMT and people living with HIV, respectively [27, 28, 43].

Each parallel stigma measure contained structural (3 items), interpersonal (9 items, 3 items per stigma source), and individual-level (6 items) stigma items (see Additional file 1, Additional file 2, Additional file 3 for measures). Responses were recorded using a 5-point Likert-type scale (1 = very low stigma, 5 = very high stigma). Intersectional HIV-related stigma scales were computed by taking the mean response for all 18 drug use, MMT, and HIV stigma items, respectively (i.e., total score), and computing mean scores for each stigma mechanism (i.e., structural, anticipated, individual) and stigma source (i.e., family, healthcare workers, other PWID) subscales. Mean stigma scores can be interpreted as 1 = very low stigma, 2 = low to moderate stigma, 3 = moderate stigma, 4 = moderate to high stigma, 5 = very high stigma.

### Statistical analyses

We computed Cronbach’s alpha (*α*) and McDonald’s omega (*ω*) for each stigma scale and subscale as a measure of internal reliability [44]. For scales with fewer than 10 items, McDonald’s omega is the preferred measure of internal reliability; while the widespread use of Cronbach’s facilitates comparisons across studies [45, 46]. Given MMT is used clinically to treat opioid dependence; we conducted a post hoc sensitivity analysis for the MMT stigma scale restricted to PWID who injected heroin in the past 6 months at baseline. Next, we calculated descriptive statistics (frequency, proportion; median interquartile range [IQR], mean, standard deviation [SD]) for all baseline study measures. We used independent *t*-test (continuous), Mann–Whitney U (ordinal), and Chi-square (categorical) test to assess whether the cohort significantly differed on study measures by urbanicity. We used Fisher’s exact test for binary variables with observed small cell sizes (< 5). Significance tests were set at *p* ≤ 0.05. We used two-tailed *p*-values on difference tests for participant characteristics (Table 1) and HIV risk and prevention behaviors (Table 2), and one-tailed *p*-values to test hypothesized differences in stigma experiences by urbanicity (Table 3).

**Table 3:** Baseline experiences of drug use, MMT, and HIV stigma overall and by urbanicity (*N* = 279)

Stigma type and mechanism	Internal reliability				Total *N* = 279	Bishkek *N* = *141*	Chuy Oblast *N* = 138	*t*_(df)_, *p*-value
	No. items	*α*	*ω*	*n*	*M*, (SD)	*M*, (SD)	*M*, (SD)	
*Drug use stigma*								
Total	18	0.81	0.77	271	3.25 (0.57)	3.30 (0.54)	3.19 (0.59)	***t***_**(269)**_** = 1.691, *p* = 0.046**
Structural	3	0.61	0.61	278	3.30 (1.10)	3.33 (1.12)	3.27 (1.08)	*t*_(276)_ = 0.440, *p* = 0.330
Anticipated	9	0.73	0.70	272	3.12 (0.63)	3.16 (0.64)	3.08 (0.62)	*t*_(270)_ = 1.151, *p* = 0.125
Family	3	0.83	0.83	273	3.18 (1.03)	3.18 (1.06)	3.17 (1.00)	*t*_(271)_ = 0.065, *p* = 0.474
Healthcare workers	3	0.69	0.69	278	3.24 (0.82)	3.33 (0.79)	3.15 (0.83)	***t***_**(276)**_** = 1.758, *p* = 0.040**
Other PWID	3	0.67	0.67	279	2.93 (0.88)	2.96 (0.91)	2.91 (0.86)	*t*_(277)_ = 0.487, *p* = 0.313
Internalized	6	0.88	0.88	279	3.39 (0.82)	3.48 (0.75)	3.30 (0.88)	***t***_**(277)**_** = 1.900, *p* = 0.029**
*MMT stigma*								
Total	18	0.80	0.80	253	3.24 (0.54)	3.25 (0.55)	3.23 (0.53)	*t*_(251)_ = 0.321, *p* = 0.374
Structural	3	0.63	0.67	262	3.04 (1.14)	3.01 (1.15)	3.06 (1.14)	*t*_(260)_ = − 0.362, *p* = 0.359
Anticipated	9	0.78	0.78	264	3.35 (0.66)	3.38 (0.68)	3.33 (0.63)	*t*_(262)_ = 0.574, *p* = 0.283
Family	3	0.68	0.69	265	3.47 (0.86)	3.51 (0.92)	3.42 (0.79)	*t*_(263)_ = 0.874, *p* = 0.191
Healthcare workers	3	0.65	0.72	270	3.35 (0.80)	3.38 (0.80)	3.32 (0.81)	*t*_(268)_ = 0.617, *p* = 0.269
Other PWID	3	0.75	0.76	273	3.22 (0.92)	3.19 (0.95)	3.24 (0.90)	*t*_(271)_ = − 0.457, *p* = 0.324
Stereotypes and prejudice	6	0.73	0.77	272	3.15 (0.64)	3.14 (0.65)	3.16 (0.64)	*t*_(270)_ = − 0.220, *p* = 0.413
*HIV stigma*								
Total	18	0.80	0.75	261	2.94 (0.54)	2.94 (0.55)	2.94 (0.52)	*t*_(259)_ = − 0.043, *p* = 0.483
Structural	3	0.60	0.61	270	2.92 (1.12)	2.94 (1.10)	2.89 (1.14)	*t*_(268)_ = 0.344, *p* = 0.365
Anticipated	9	0.81	0.78	272	3.11 (0.69)	3.10 (0.72)	3.12 (0.66)	*t*_(270)_ = − 0.026, *p* = 0.490
Family	3	0.89	0.89	273	2.81 (1.07)	2.85 (1.09)	2.77 (1.05)	*t*_(271)_ = 0.640, *p* = 0.261
Healthcare workers	3	0.79	0.80	278	3.08 (0.89)	3.07 (0.91)	3.10 (0.88)	*t*_(276)_ = − 0.336, *p* = 0.369
Other PWID	3	0.70	0.71	279	3.43 (0.84)	3.40 (0.86)	3.47 (0.82)	*t*_(277)_ = − 0.687, *p* = 0.246
Stereotypes and prejudice	6	0.64	0.66	277	2.68 (0.56)	2.67 (0.56)	2.70 (0.57)	*t*_(275)_ = − 0.395, *p* = 0.693
Internalized (*n* = 57)*	6	0.88	0.89	57	2.78 (0.88)	2.62 (0.85)	3.15 (0.86)	***t***_**(55)**_** = − 2.188, *p* = 0.033**

Mean stigma scores can be interpreted as 1 = very low stigma, 2 = low to moderate stigma, 3 = moderate stigma, 4 = moderate to high stigma, 5 = very high stigma

Statistically significant differences are in bold

*Internalized stigma items were only asked of PWID who reported being diagnosed with HIV at baseline. Internalized HIV stigma items are not included in the total 18-item HIV stigma score

## Results

### Study population

KISS successfully enrolled a geographically diverse cohort of 279 PWID (141 from Bishkek [50.5%], 138 from Chuy Oblast [49.5%]). We enrolled 204 (73.1%) PWID via modified time location sampling and 75 (26.9%) PWID not actively engaged in HIV prevention services via snowball sampling. Only 10 (8.9%) of the 112 PWID approached by outreach workers in Bishkek using our modified venue-based time location sampling method declined study participation. No PWID approached by outreach workers in Chuy Oblast declined participation.

### Baseline demographics and substance use characteristics

As given in Table 1, the median age was 40 years old (IQR 35–46); 134 participants (48.0%) were age 20–39. The majority of the sample identified as men (75.3%, *n* = 210), ethnically Russian (53.8%, *n* = 150), most had a high school education (38.0%, *n* = 106) or less (35.5%, *n* = 99). Significantly more unstable housing was reported among PWID in Bishkek (51.8%, *n* = 73) compared to Chuy Oblast (37.0%, *n* = 51; *p* = 0.013). Sixty-six PWID had a seropositive rapid HIV test at baseline, among whom five were newly diagnosed. Of the 61 PWID previously diagnosed with HIV, 57 reported their serostatus in the baseline survey, while 4 disclosed their serostatus after the survey was completed meaning HIV care and treatment data were not collected. PWID recruited from Bishkek (69.7%, *n* = 46) were significantly more likely to be living with HIV compared to Chuy Oblast (30.3%, *n* = 20; *p* = 0.002).

Median age at first injection was 20 years old (IQR 17–24). In the past 6 months, most participants injected once a week (22.9%, *n* = 64) or more (27.2%, *n* = 76) with 10% (*n* = 23) of participants injecting daily. Drugs injected by most PWID in the past 6 months were heroin (90.2%, *n* = 240), diphenhydramine (60.7%, *n* = 142), and bath salts (21.5%, *n* = 39). Stimulant and benzodiazepine injection was less common, though significantly more PWID injected benzodiazepines in Bishkek (12.8%, *n* = 18) than Chuy Oblast (6.1%, *n* = 6; *p* = 0.019). Poly-drug use was common with 181 participants (64.9%) reporting they injected more than one drug type in the past 6 months, and approximately one-third of the sample reported hazardous drinking (33.9%).

### HIV risk and prevention service utilization

As shown in Table 2, 16.5% (*n* = 46) of the sample reported sharing injection equipment in the past 6 months, 35.9% (*n* = 99) injected in public, 34.4% (*n* = 95) reused a cooker or filter, and 31.9% (*n* = 89) had drugs to inject but could not access a sterile syringe. Self-reported lifetime (67.4%, *n* = 188) and past 6 months (10.0%, *n* = 28) accidental overdose was high. In the past 6 months, the majority of participants (*n* = 218, 78.1%) were sexually active, among whom 68.3% (*n* = 149) reported any condomless vaginal or anal sex and 70.6% (*n* = 154) reported having multiple sexual partners. Significantly more PWID in Bishkek (13.9%, *n* = 14) reported engaging in sex work in the past 6 months compared to Chuy Oblast (2.8%, *n* = 3; *p* = 0.003). Over one-third of the sample (34.1%, *n* = 95) reported not accessing SSP services in the past 6 months, and only 8.2% (*n* = 23) reported current MMT use. Among the 218 PWID who tested HIV-seronegative or who were newly diagnosed at baseline, 61.0% (*n* = 133) had an HIV test in the past 12 months and 18.8% (*n* = 41) had heard of PrEP. No PWID reported ever taking PrEP (data not shown). Among the 57 PWID living with HIV who reported knowing their HIV-positive serostatus at baseline, more than 90% self-reported having at least one HIV care visit in the past 6 months (*n* = 53), were taking ART (*n* = 56) and reported ≥ 80% ART adherence (*n* = 52). Only 38.6% (*n* = 22) reported perfect ART adherence.

### HIV-related intersectional stigma

Measures of internal reliability was strong for the total mean drug use, MMT, and HIV stigma (≥ 0.80), and most anticipated and individual-level stigma mechanism and stigma source subscales (*α* = 0.73–0.89). Internal reliability was acceptable (*α* = 0.60–0.63) for structural stigma subscales (Table 3). We observed similar internal reliability estimates for MMT stigma when analyses were restricted to the 240 PWID who injected heroin in the past 6 months (see Additional file 4).

On average, PWID enrolled in the KISS cohort reported moderate levels of HIV-related intersectional stigma, with participants having higher total mean stigma scores for drug use (*M* = 3.25, SD = 0.057) and MMT stigma (*M* = 3.24, SD = 0.54) than HIV stigma (*M* = 2.94, SD = 0.055). We observed similar levels of total mean MMT stigma (*M* = 3.23, SD = 0.55) in our sensitivity analyses restricted to the 240 PWID who injected heroin in the past 6 months. Contrary to our hypothesis, total mean stigma scores for drug use stigma were significantly higher among PWID from Bishkek (*M* = 3.30, SD = 0.54) compared to Chuy Oblast (*M* = 3.19, SD = 0.59; *t*_(269)_ = 1.691, *p* = 0.046). This difference was largely accounted for by significantly higher mean scores for anticipated drug use stigma from healthcare workers (*t*_(276)_ = 1.758, *p* = 0.040) and internalized drug use stigma (*t*_(277)_ = 1.900, *p* = 0.029) among PWID in Bishkek compared to Chuy Oblast (see Table 3). Counter to our hypotheses, significant differences by urbanicity were not observed for MMT (*t*_(251)_ = 0.321, *p* = 0.374) or HIV stigma (*t*_(259)_ = − 0.043, *p* = 0.483) in this sample. While not hypothesized, internalized HIV stigma among the 57 PWID reporting their HIV-seropositive status at baseline was significantly higher among PWID from Chuy Oblast (*M* = 3.15, SD = 0.86) compared to Bishkek (*M* = 2.62, SD = 0.85, *t*_(55)_ = − 2.188, *p* = 0.033).

All three stigma source hypotheses were supported. Mean anticipated drug use stigma scores was highest for healthcare workers (*M* = 3.24, *SD* = 0.82) than other stigma sources (family *M* = 3.18, *SD* = 1.03, other PWID *M* = 2.93, *SD* = 0.88). Mean anticipated MMT stigma scores was highest for family members (*M* = 3.47, *SD* = 0.86) than other stigma sources (healthcare workers *M* = 3.35, *SD* = 0.80, other PWID *M* = 3.22, *SD* = 0.92). Mean anticipated HIV stigma scores was highest for other PWID (*M* = 3.43, *SD* = 0.84) than other stigma sources (family *M* = 2.81, *SD* = 1.07, healthcare workers *M* = 3.08, *SD* = 0.89).

### Retention rates for visit 2

Among the 204 participants eligible for a second study visit, 189 (92.6%) were retained on study protocol. Most completed the follow-up survey at month 3 (*n* = 173, 84.9%). Nine participants (4.4%) were located but did not complete a survey. Among whom, two participants’ survey data was not recorded due to technical issues with the survey link, two declined to complete the survey due to COVID, and five were detained by the police. Seven participants (3.4%) were confirmed deceased. Stated cause of death were overdose (*n* = 2), COVID-19 (*n* = 2), and health conditions likely exacerbated by drug use and related healthcare barriers (heart failure, pulmonary arrest, and brain aneurysm). Only 15 participants were lost to follow-up (7.4%), among whom eight had a disconnected phone, four had invalid contact information, and three were not located with valid contact information.

## Discussion

The KISS cohort successfully enrolled and retained a diverse sample of PWID, including harder-to-reach PWID not engaged in HIV prevention services. PWID from Bishkek and Chuy Oblast were generally similar, though higher rates of unstable housing, sex work, and stimulant use in Bishkek highlight important factors that may heighten vulnerability to HIV transmission and poorer health outcomes in an urban environment. Despite the availability of numerous HIV prevention services targeting PWID in Kyrgyzstan, we observed suboptimal rates of service utilization in the KISS cohort with approximately one-third of the sample not meeting HIV testing guidelines or accessing SSP services. Our data further highlight opportunities to improve low uptake and poor retention of PWID in MMT care, responding to a 21% national decline in MMT utilization since 2016 [47], despite similar rates of injection drug use in recent years [4]. Finally, opportunities to strengthen ART adherence among PWID living with HIV currently engaged in HIV care to prevent medication resistance, optimize health outcomes, and reduce onward transmission are warranted [48].

To our knowledge KISS is the first cohort study to systematically examine HIV-related intersectional stigma in relation to the health and HIV prevention needs of PWID, and it does so in a region disproportionately impacted by this phenomenon and burdened by an expanding HIV epidemic. Prior work observed lower access to HIV prevention services in rural areas in Kyrgyzstan and EECA regions which may lead to greater anticipated stigma in rural areas if such services were accessed [6, 10]. One plausible explanation why we observed similar levels of MMT and HIV stigma among urban and rural participants in the KISS cohort is that participants reported similarly low levels of engagement in SSP, MMT, and HIV testing services across urbanicity. Future work should assess whether higher rates of unstable housing, sex work, and stimulant use in the urban environment partially explain the significantly higher levels of drug use stigma observed among PWID from Bishkek. We observed higher engagement in HIV care in Bishkek and Chuy Oblast. Notably, Bishkek is home to the largest provider of HIV services in Kyrgyzstan, the Republican AIDS Center. Future work should explore whether factors such as lower access to high quality HIV care and more interconnected social networks in rural settings account for higher levels of internalized HIV stigma among those living in Chuy Oblast.

Compared to our prior work examining HIV-related intersectional stigma via parallel measures among people who use drugs in the United States who are living with HIV or engaged in MMT [27], participants in the KISS cohort reported higher mean levels of anticipated drug use, MMT, and HIV stigma [26], but similar levels of internalized drug use stigma [28]. These data build on previous stigma research in the region by providing insights into how PWID experience intersectional HIV-related stigma. Notably, prior research emphasized structural manifestations of stigma such as police harassment, being registered in government systems, and being denied housing and employment as substantial barriers to engagement in HIV prevention services [17–19, 25, 42, 49]. Our data suggest that structural stigma concerns were on average higher for drug use and MMT than HIV among PWID in the KISS cohort. Similarly, our data reinforce previous observations that healthcare workers, family, and other PWID are important stigma reduction targets. Uniquely, findings from the KISS cohort help to identify which stigma sources may have greater impact on the health, treatment, and prevention needs of PWID. Through future work leveraging advanced quantitative techniques (e.g., latent profile analysis, moderated mediation) and KISS cohort data should facilitate a deeper understanding of how and among whom we can optimize stigma reduction interventions across multiple levels to improve engagement in HIV prevention services.

## Strengths and weaknesses

The KISS cohort study demonstrated the capacity to reach, enroll, and retain, PWID in a high-risk international setting. Compared to other well-established PWID cohort studies, the KISS cohort is smaller in scope, and limited in the total time and proportion of participants that were followed prospectively. However, the cohort represents PWID in the EECA region where the HIV epidemic is rapidly expanding and efforts to improve the uptake of evidence-based HIV prevention services in urban and rural settings are urgently needed. By utilizing two non-random purposive sampling frameworks, we further enhance our understanding of PWID not engaged in existing HIV services. As with any study examining socially stigmatized behaviors via self-report, social desirability bias is a potential limitation. However, we observed high response rates on injection and sexual risk items, and variation in mean levels of stigma reported across stigma types, mechanisms, and sources strengthening our confidence in these data. Finally, data reported in this manuscript are cross-sectional and descriptive in nature, meaning causality cannot be inferred without future prospective analyses.

## Conclusions

With an emphasis on evaluating the impact of HIV-related intersectional stigma on HIV risk and prevention service utilization among PWID in Kyrgyzstan, the KISS cohort responds to the need to expand our understanding of stigma as a fundamental driver of rising HIV transmission and AIDS mortality in the EECA region. This cohort provides the opportunity to evaluate multidimensional relationships between drug use, MMT, and HIV stigma among PWID in urban and rural environments. Ultimately, the KISS cohort provides a unique and timely opportunity to facilitate a deeper, multidimensional, understanding of how to optimize stigma reduction efforts to mitigate stigma-related harms and advance the utility of extant HIV prevention efforts in the region.

## Supplementary Information

**Additional file 1**. Drug use stigma measure: English, Russian

**Additional file 2**. Methadone maintenance treatment stigma measure: English, Russian

**Additional file 3**. HIV stigma measure: English, Russian

**Additional file 4**. Methadone maintenance treatment stigma scale post hoc sensitivity analysis

## Data Availability

The datasets analyzed during the current study are available from the corresponding author on reasonable request.

## Abbreviations

AIDS: Acquired immunodeficiency syndrome
ART: Antiretroviral therapy
AUDIT: Alcohol use disorders identification test
COVID-19: Coronavirus disease 2019
EECA: Eastern Europe and Central Asia
GLORI: GLObal Research Institute
HIV: Human immunodeficiency virus
IRB: Institutional Review Board
IQR: Interquartile range
PrEP: Pre-exposure prophylaxis
KISS: Kyrgyzstan InterSectional Stigma
MMT: Methadone maintenance treatment
PWID: People who inject drugs
SD: Standard deviation
SSP: Syringe service programs
WHO: World Health Organization
