# Supplementary material for: Cohort profile: the Kyrgyzstan InterSectional Stigma (KISS) injection drug use cohort study
Source: Harm Reduct J. 2022 May 25;19:53. doi: 10.1186/s12954-022-00633-5 (PMC9131652; doi:10.1186/s12954-022-00633-5)
Supplement: Supplementary file 1 — Additional file 1. Drug use stigma measure: English, Russian [file 12954_2022_633_MOESM1_ESM.docx]

**Additional file 1 (Drug Use Stigma Measure: English, Russian)**

**Supplementary Material for the Article:**

Cohort Profile: The Kyrgyzstan InterSectional Stigma (KISS) Injection Drug Use Cohort Study

**Author Names and Affiliations:**

Laramie R. Smith^1^, Natalia Shumskaia^2^, Ainura Kurmanalieva^2^, Thomas L. Patterson^3^, Dan Werb^1,4^, Anna Bluym^1^, Angel B. Algarin^1^, Samantha Yeager^1^, and Javier Cepeda^5^

^1^ Division of Infectious Diseases and Global Public Health, University of California, San Diego, La Jolla, CA, USA

^2^ AIDS Foundation – East West in the Kyrgyz Republic, Bishkek, Kyrgyzstan

^3^ Department of Psychiatry, University of California, San Diego, La Jolla, CA, USA

^4^ Centre on Drug Policy Evaluation, St. Michael’s Hospital, Toronto, Canada

^5^ Department of Epidemiology, Johns Hopkins Bloomberg School of Public Health, Baltimore, MD, USA

**Corresponding Author:**

Laramie R Smith, PhD. Associate Professor. Division of Infectious Diseases and Global Public Health, Department of Medicine, University of California San Diego, 9500 Gilman Drive, Mail Code 0507, La Jolla, CA 92093-0507. Phone: +1 858-822-1462. E-mail: [laramie@ucsd.edu](mailto:laramie@ucsd.edu)

**This material supplements, but does not replace, the peer-reviewed paper in the *Harm Reduction Journal* and reflects partial supplemental material previously published in *Addiction*.**

**Multilevel Drug Use Stigma Mechanism Scale (mDU-SMS)**

**The original scale:** This scale has been adapted from the original Substance Use Stigma Mechanisms Scale. The initial validation of this measure was conducted in the United States (U.S.) with substance-using populations and was published in *Drug and Alcohol Dependence* and *Addiction,* cited below. The original SU-SMS was co-developed by Laramie R. Smith, Ph.D. and Valerie A. Earnshaw, Ph.D.

**The current scale:** The Multilevel Drug Use Stigma Mechanisms Scale (mDU-SMS) was revised to be implemented with persons who inject drugs (PWID) specifically. As such, the term ‘substance use’ was replaced with a more precise term, ‘drug use’. The current scale was revised by Laramie R. Smith, Ph.D.

The current scale includes the internalized stigma subscale to assess individual-level experiences of stigma, and anticipated stigma subscales from two stigma sources (family members, healthcare workers) to assess interpersonal-level manifestations of stigma as measured in the original scale. The current scale includes a third source of anticipated stigma identified in the extant literature as influencing HIV prevention outcomes among PWID (i.e. other persons who inject drugs). This third stigma source replaces ‘employers’ as the third anticipated stigma source subscale assessed in the original validation of the scale in the U.S. context.

The current version of the scale also assesses experiences of anticipated structural stigma that were not assessed in the original SU-SMS. This includes the addition a three-item subscale that measures how concerned the participant is that they will experience structural or system-level consequences because they use drugs. These anticipated structural consequences were informed by the extant literature on structural manifestations of stigma in the Eastern European Central Asia context (i.e., police harassment, registration in government-based systems as a person who uses drugs, and being denied housing or employment).

**Original Scale Citations:**

Smith LR, Earnshaw VA, Copenhaver MM, Cunningham C. Substance use stigma: Reliability and validity of a theory-based scale for substance-using populations. 2016. *Drug Alcohol Dependence*; 162:34-43.

doi: 10.1016/j.drugalcdep.2016.02.019.

Smith LR, Mittal ML, Wagner K, Copenhaver MM, Cunningham CO, Earnshaw VA. Factor structure, internal reliability and construct validity of the Methadone Maintenance Treatment Stigma Mechanisms Scale (MMT-SMS). 2019. *Addiction*. doi: 10.1111/add.14799.

**Intended use:** The mDU-SMS may be administered to people who inject drugs, including those who are out-of-treatment, non-treatment seeking, treatment-seeking, and in-treatment for substance use disorders.

**Scoring:** All responses are given on a 5-point Likert-type scale, with higher scores indicating greater endorsement of substance use stigma. Structural (3 items), Anticipated (9 items), and Internalized (6 items) scales can be created by taking the average of the item responses given for each stigma mechanism respectively. Stigma source sub-scales can be created for Anticipated stigma by taking the average responses given for the family members (3 items), healthcare workers (3 items), and other persons who inject drugs (3 items), item responses respectively.

**English: Multilevel Drug Use Stigma Mechanism Scale (mDU-SMS)**

**Instructions:** These questions will ask about how you might be treated in the future because of your drug use history. Please select one response option for each question. Do not spend too much time considering your answer. Your first impression is usually best.

**ANTICIPATED STRUCTURAL STIGMA** (header can be omitted in the survey)

|  |  | Not at all | A little bit | Somewhat | Quite a bit | Extremely |
| --- | --- | --- | --- | --- | --- | --- |
|  | How concerned are you that the police will harass you because you use drugs? | 1 | 2 | 3 | 4 | 5 |
|  | How concerned are you that you will be registered in the system as someone who uses drugs if you seek treatment? | 1 | 2 | 3 | 4 | 5 |
|  | How concerned are you that you will be denied housing or employment because you use drugs? | 1 | 2 | 3 | 4 | 5 |

**ANTICIPATED INTERPERSONAL STIGMA** (header can be omitted in survey)

How likely is it that people will treat you in the following ways in the future because of your drug use history?

|  |  | Very unlikely | Unlikely | Neither unlikely nor likely | Likely | Very likely |
| --- | --- | --- | --- | --- | --- | --- |
| 4. | Family members will think that I cannot be trusted. | 1 | 2 | 3 | 4 | 5 |
| 5. | Family members will look down on me. | 1 | 2 | 3 | 4 | 5 |
| 6. | Family members will treat me differently. | 1 | 2 | 3 | 4 | 5 |
| 7. | Healthcare workers will not listen to my concerns. | 1 | 2 | 3 | 4 | 5 |
| 8. | Healthcare workers will think that I’m pill shopping, or trying to con them into giving me prescription medications to get high or sell. | 1 | 2 | 3 | 4 | 5 |
| 9. | Healthcare workers will give me poor care. | 1 | 2 | 3 | 4 | 5 |
| 10. | Other people who inject drugs will think I’m weak (because I use drugs). | 1 | 2 | 3 | 4 | 5 |
| 11. | Other people who just drugs will not support me (because I use drugs). | 1 | 2 | 3 | 4 | 5 |
| 12. | Other people who use drugs won’t trust me (because I use drugs). | 1 | 2 | 3 | 4 | 5 |

**INTERNALIZED STIGMA** (header can be omitted in survey)

**Instructions:** These next statements reflect the way some people have said they felt about their drug use history. Please indicate how much you agree with each statement. Do not spend too much time considering your answer. Your first impression is usually best.

How do you feel about your own drug use?

|  |  | Strongly disagree | Disagree | Neither disagree nor agree | Agree | Strongly agree |
| --- | --- | --- | --- | --- | --- | --- |
| 13. | Having used drugs makes me feel like I’m a bad person. | 1 | 2 | 3 | 4 | 5 |
| 14. | I feel I’m not as good as others because I used drugs. | 1 | 2 | 3 | 4 | 5 |
| 15. | I feel ashamed of having used drugs. | 1 | 2 | 3 | 4 | 5 |
| 16. | I think less of myself because I used drugs. | 1 | 2 | 3 | 4 | 5 |
| 17. | Having used drugs makes me feel unclean. | 1 | 2 | 3 | 4 | 5 |
| 18. | Having used drugs is disgusting to me. | 1 | 2 | 3 | 4 | 5 |

**Russian: Multilevel Drug Use Stigma Mechanism Scale (mDU-SMS)**

**Многоуровневая шкала для изучения механизма стигматизации, связанной**

**с употреблением наркотиков (mDU-SMS)**

**Предназначена для использования**: Шкалу mDU-SMS можно администрировать среди людей, употребляющих инъекционные наркотики, в том числе тех, кто не проходит лечение, не обращается за лечением, либо обращается за лечением и проходит лечение в связи с расстройством, связанным с употреблением психоактивных веществ.

**Оценка:** Все ответы даются по 5-балльной шкале вроде шкалы Лайкерта, где более высокие баллы указывают на большее подтверждение стигматизации, связанной с употреблением психоактивных веществ. Балл для оценки структурной (3 вопроса), ожидаемой/ прогнозируемой (9 вопросов) и интернализованной стигмы (внутренней стигмы/ самостигмы) (6 вопросов) можно определить, взяв среднее значение ответов, полученных соответственно по каждому механизму стигмы. Под-шкалы источников стигмы могут быть созданы для ожидаемой стигмы, взяв среднее значение ответов, полученных соответственно на вопросы касательно членов семьи (3 пункта), медицинских работников (3 пункта) и других лиц, употребляющих инъекционные наркотики (3 пункта).

**Предназначена для использования**: Шкалу mDU-SMS можно администрировать среди людей, употребляющих инъекционные наркотики, в том числе тех, кто не проходит лечение, не обращается за лечением, либо обращается за лечением и проходит лечение в связи с расстройством, связанным с употреблением психоактивных веществ.

**Оценка:** Все ответы даются по 5-балльной шкале вроде шкалы Лайкерта, где более высокие баллы указывают на большее подтверждение стигматизации, связанной с употреблением психоактивных веществ. Балл для оценки структурной (3 пункта), ожидаемой/ прогнозируемой (9 пунктов) и интернализованной стигмы (внутренней стигмы/ самостигмы) (6 пунктов) можно определить, взяв среднее значение ответов, данных соответственно по каждому механизму стигмы. Под-шкалы источников стигмы могут быть созданы для ожидаемой стигмы можно определить, взяв среднее значение ответов, данных соответственно на вопросы касательно членов семьи (3 пункта), медицинских работников (3 пункта) и других лиц, употребляющих инъекционные наркотики (3 пункта).

**Инструкция:** Следующие вопросы коснутся Вашего восприятия того, как к Вам могут отнестись в будущем с учетом истории Вашего потребления наркотических веществ. Пожалуйста, выберите один вариант ответа на каждый вопрос. Не раздумывайте долго над ответом. Первый возникший ответ зачастую - лучший.

**ОЖИДАЕМАЯ СТРУКТУРНАЯ СТИГМА** (Заголовок можно опустить при администрировании опроса)

|  |  | Совсем не обеспокоен | Немного | В какой-то степени | Достаточно сильно | Чрезвычайно |
| --- | --- | --- | --- | --- | --- | --- |
|  | Насколько Вы обеспокоены тем, что милиция будет преследовать Вас в связи с Вашим потреблением наркотиков? | 1 | 2 | 3 | 4 | 5 |
|  | Насколько Вы обеспокоены тем, что, если Вы обратитесь за лечением, Вы будете зарегистрированы как человек, употребляющий наркотики? | 1 | 2 | 3 | 4 | 5 |
|  | Насколько Вы обеспокоены тем, что Вам откажут в жилье или трудоустройстве в связи с Вашим потреблением наркотиков? | 1 | 2 | 3 | 4 | 5 |

**ОЖИДАЕМАЯ МЕЖЛИЧНОСТНАЯ СТИГМА** (Заголовок можно опустить при администрировании опроса)

Насколько, по Вашему мнению, велика вероятность того, что к Вам могут отнестись определенным образом в связи с тем, что у Вас есть история потребления наркотических веществ?

|  |  | Очень маловероятно | Маловероятно | Затрудняюсь ответить: ни маловероятно, ни вероятно | Вероятно | Очень вероятно |
| --- | --- | --- | --- | --- | --- | --- |
| 4. | Члены семьи будут считать, что я не достоен/не достойна доверия | 1 | 2 | 3 | 4 | 5 |
| 5. | Члены семьи буду смотреть на меня свысока. | 1 | 2 | 3 | 4 | 5 |
| 6. | Члены семьи будут относиться ко мне по-другому. | 1 | 2 | 3 | 4 | 5 |
| 7. | Медицинские работники не будут прислушиваться к моим проблемам и опасениям. | 1 | 2 | 3 | 4 | 5 |
| 8. | Медицинские работники будут думать, что я пытаюсь заполучить таблетки, или обмануть их, чтобы мне выписали медикаменты по рецепту, которыми я потом воспользуюсь, чтобы получить кайф или продать. | 1 | 2 | 3 | 4 | 5 |
| 9. | Медицинские работники будут плохо за мной ухаживать. | 1 | 2 | 3 | 4 | 5 |
| 10. | Другие люди, употребляющие наркотические вещества инъекционно, будут считать меня слабым(-ой) (потому что я употребляю наркотики). | 1 | 2 | 3 | 4 | 5 |
| 11. | Другие люди, употребляющие наркотические вещества инъекционно, не будут поддерживать меня (потому что я употребляю наркотики). | 1 | 2 | 3 | 4 | 5 |
| 12. | Другие люди, употребляющие наркотические вещества инъекционно, не будут доверять мне (потому что я употребляю наркотики). | 1 | 2 | 3 | 4 | 5 |

**ВНУТРЕННЯЯ СТИГМА (САМОСТИГМА)** (Заголовок можно опустить при администрировании опроса)

**Инструкции**: Следующие утверждения отражают чувства, которые, как сообщили некоторые люди, они испытали относительно своей истории употребления наркотиков. Пожалуйста, укажите, насколько Вы согласны с каждым утверждением. Не тратьте слишком много времени на обдумывание своего ответа. Ваше первое впечатление - зачастую самое лучшее.

Как Вы относитесь к истории своего потребления наркотических веществ?

|  |  | Совершенно не согласен | Не согласен | Нейтрален: ни не согласен, ни согласен | Согласен | Полностью согласен |
| --- | --- | --- | --- | --- | --- | --- |
| 13. | Употребление наркотиков заставляет меня чувствовать себя плохим человеком. | 1 | 2 | 3 | 4 | 5 |
| 14. | Я чувствую себя хуже других в связи с моим потреблением наркотиков. | 1 | 2 | 3 | 4 | 5 |
| 15. | Я испытываю стыд за свое употребление наркотиков. | 1 | 2 | 3 | 4 | 5 |
| 16. | Я думаю о себе хуже в связи с употреблением наркотиков. | 1 | 2 | 3 | 4 | 5 |
| 17. | После употребления наркотиков я чувствую себя нечистым. | 1 | 2 | 3 | 4 | 5 |
| 18. | Мне противно, что я употреблял наркотики. | 1 | 2 | 3 | 4 | 5 |
