# Supplementary material for: Cohort profile: the Kyrgyzstan InterSectional Stigma (KISS) injection drug use cohort study
Source: Harm Reduct J. 2022 May 25;19:53. doi: 10.1186/s12954-022-00633-5 (PMC9131652; doi:10.1186/s12954-022-00633-5)
Supplement: Supplementary file 2 — Additional file 2. Methadone maintenance treatment stigma measure: English, Russian [file 12954_2022_633_MOESM2_ESM.docx]

**Additional file 2 (Methadone Maintenance Treatment Stigma Measure: English, Russian)**

**Supplementary Material for the Article:**

Cohort Profile: The Kyrgyzstan InterSectional Stigma (KISS) Injection Drug Use Cohort Study

**Author Names and Affiliations:**

Laramie R. Smith^1^, Natalia Shumskaia^2^, Ainura Kurmanalieva^2^, Thomas L. Patterson^3^, Dan Werb^1,4^, Anna Bluym^1^, Angel B. Algarin^1^, Samantha Yeager^1^, and Javier Cepeda^5^

^1^ Division of Infectious Diseases and Global Public Health, University of California, San Diego, La Jolla, CA, USA

^2^ AIDS Foundation – East West in the Kyrgyz Republic, Bishkek, Kyrgyzstan

^3^ Department of Psychiatry, University of California, San Diego, La Jolla, CA, USA

^4^ Centre on Drug Policy Evaluation, St. Michael’s Hospital, Toronto, Canada

^5^ Department of Epidemiology, Johns Hopkins Bloomberg School of Public Health, Baltimore, MD, USA

**Corresponding Author:**

Laramie R Smith, PhD. Associate Professor. Division of Infectious Diseases and Global Public Health, Department of Medicine, University of California San Diego, 9500 Gilman Drive, Mail Code 0507, La Jolla, CA 92093-0507. Phone: +1 858-822-1462. E-mail: [laramie@ucsd.edu](mailto:laramie@ucsd.edu)

**This material supplements, but does not replace, the peer-reviewed paper in the *Harm Reduction Journal* and reflects partial supplemental material previously published in *Addiction*.**

**Multilevel Methadone Maintenance Treatment Stigma Mechanism Scale (mMMT-SMS)**

**The original scale:** This scale has been adapted from the original Methadone Maintenance Treatment (MMT) Stigma Mechanisms Scale. The initial validation of this measure was conducted in the United States (U.S.) with substance-using populations enrolled in MMT and was published in *Addiction,* cited below. The original MMT-SMS was co-developed by Laramie R. Smith, Ph.D. and Valerie A. Earnshaw, Ph.D.

**The current scale:** The Multilevel Methadone Maintenance Treatment Stigma Mechanisms Scale (mMMT-SMS) was revised to be implemented with persons who inject drugs regardless of their MMT use history. As such, the phrase ‘because you receive methadone’ was replaced with the phrase, ‘if you were to take methadone’. The current scale was revised by Laramie R. Smith, Ph.D.

The current scale includes the anticipated stigma subscales from two stigma sources (family members, healthcare workers) to assess interpersonal-level manifestations of stigma as measured in the original scale. The current scale includes a third source of anticipated stigma identified in the extant literature as influencing HIV prevention outcomes among PWID (i.e. other persons who inject drugs). This third stigma source replaces ‘employers’ as the third anticipated stigma source subscale assessed in the original validation of the scale in the U.S. context.

The current version of the scale also assesses experiences of anticipated structural stigma that were not assessed in the original MMT-SMS. This includes the addition a three-item subscale that measures how concerned the participant is that they will experience structural or system-level consequences if they take methadone or seek methadone treatment. These anticipated structural consequences were informed by the extant literature on structural manifestations of stigma in the Eastern European Central Asia context (i.e., police harassment, registration in government-based systems as a person who takes MMT, and being denied housing or employment).

Finally, the current scale includes six new items to assess individual-level experiences of stigma towards MMT among PWID by assessing the degree to which PWID endorse stereotypes (3-items) and prejudice (3-items) about people who take methadone. These items were adapted from previous HIV stereotype and prejudice items developed by Laramie R. Smith, Ph.D. and Valarie A. Earnshaw, Ph.D., and the content of each item was tailored to reflect MMT stereotypes and prejudice towards MMT observed in the extant literature.

**Original Scale Citation:**

Smith LR, Mittal ML, Wagner K, Copenhaver MM, Cunningham CO, Earnshaw VA. Factor structure, internal reliability and construct validity of the Methadone Maintenance Treatment Stigma Mechanisms Scale (MMT-SMS). 2019. *Addiction*. doi: 10.1111/add.14799.

**Intended use:** The mMMT-SMS was developed for use in a diverse range of people who inject drugs, who are expected to be aware of social stigma towards MMT regardless of their personal MMT treatment history. It may be adapted for persons experienced with other forms of medication-assisted therapy for opioid use disorders accordingly.

**Scoring:** All responses are given on a 5-point Likert-type scale, with higher scores indicating greater endorsement of MMT stigma. Structural (3 items), Anticipated (9 items), and Stereotypes/Prejudice (6 items) scales can be created by taking the average of the item responses given for each stigma mechanism respectively. Stigma source sub-scales can be created for Anticipated stigma by taking the average responses given for the family members (3 items), healthcare workers (3 items), and other persons who inject drugs (3 items), item responses respectively.

**English: Multilevel MMT Stigma Mechanism Scale (mMMT-SMS)**

**Instructions:** Methadone is a medication that can be taken to treat opioid dependence. Methadone is a synthetic opioid agonist that eliminates withdrawal symptoms and relieves drug cravings by acting on opioid receptors in the brain-the same receptors that other opioids such as heroin, morphine, and opioid pain medications activate. Although it occupies and activates these opioid receptors, it does so more slowly than other opioids and, in an opioid dependent person, treatment does do not produce euphoria. **These questions will ask about how you might be treated in the future if you were taking methadone. Please select one response option for each question. Do not spend too much time considering your answer. Your first impression is usually best.**

**ANTICIPATED STRUCTURAL STIGMA** (header can be omitted in the survey)

|  |  | Not at all | A little bit | Somewhat | Quite a bit | Extremely |
| --- | --- | --- | --- | --- | --- | --- |
| 1. | How concerned are you that the police will harass you if you take methadone? | 1 | 2 | 3 | 4 | 5 |
| 2. | How concerned are you that you will be registered in the system as someone who uses methadone if you seek treatment? | 1 | 2 | 3 | 4 | 5 |
| 3. | How concerned are you that you will be denied housing or employment because you take methadone? | 1 | 2 | 3 | 4 | 5 |

**ANTICIPATED INTERPERSONAL STIGMA** (header can be omitted in survey)

How likely is it that people will treat you in the following ways in the **future** if you were to take methadone**?**

|  |  | Very unlikely | Unlikely | Neither unlikely nor likely | Likely | Very Likely |
| --- | --- | --- | --- | --- | --- | --- |
| 4. | Family members will think that I’m still a drug user. | 1 | 2 | 3 | 4 | 5 |
| 5. | Family members will not support my methadone treatment. | 1 | 2 | 3 | 4 | 5 |
| 6. | Family members will think that I cannot recover. | 1 | 2 | 3 | 4 | 5 |
| 7. | Healthcare workers will think I’m still a drug user. | 1 | 2 | 3 | 4 | 5 |
| 8. | Healthcare workers will give me poor care. | 1 | 2 | 3 | 4 | 5 |
| 9. | Healthcare workers will not prescribe me medication that I need. | 1 | 2 | 3 | 4 | 5 |
| 10. | Other people who inject drugs will think I’m weak (if I take methadone). | 1 | 2 | 3 | 4 | 5 |
| 11. | Other people who use drugs will not support me (if I take methadone). | 1 | 2 | 3 | 4 | 5 |
| 12. | Other people who use drugs won’t trust me (if I take methadone). | 1 | 2 | 3 | 4 | 5 |

**STEREOTYPES AND PREJUDICE** (header can be omitted in survey)

**Instructions:** These next statements reflect the way some people have said they felt about people who take methadone. Please indicate much do you agree with each statement. Do not spend too much time considering your answer. Your first impression is usually best.

How do you **feel** about people who take methadone?

|  |  | Strongly disagree | Disagree | Neither disagree nor agree | Agree | Strongly agree |
| --- | --- | --- | --- | --- | --- | --- |
| 13. | People who take methadone are replacing one drug for another. | 1 | 2 | 3 | 4 | 5 |
| 14. | People who take methadone cannot be trusted. | 1 | 2 | 3 | 4 | 5 |
| 15. | People who take methadone are not in control of their own life. | 1 | 2 | 3 | 4 | 5 |
| 16. | People who take methadone make me feel uncomfortable. | 1 | 2 | 3 | 4 | 5 |
| 17. | People who take methadone make me feel hopeful. | 1 | 2 | 3 | 4 | 5 |
| 18. | People who take methadone make me feel angry | 1 | 2 | 3 | 4 | 5 |

**Russian: Multilevel MMT Stigma Mechanism Scale (mMMT-SMS)**

**Многоуровневая шкала для изучения механизма стигматизации, связанной с поддерживающей терапией с применением метадона (MMT)**

**Предназначена для использования:** Шкала mMMT-SMS был разработан для использования среди широкого круга людей, употребляющих инъекционные наркотики, которые, как ожидается, будут осведомлены о социальной стигматизации по отношению к MMT, независимо от их личной истории лечения с MMT. Шкала может быть адаптирована для лиц, имеющих опыт применения других форм медикаментозной терапии расстройств, связанных с употреблением опиоидов.

**Оценка:** Все ответы даются по 5-балльной шкале вроде шкалы Лайкерта, где более высокие баллы указывают на большее подтверждение стигматизации, связанной с ММТ. Балл для оценки структурной (3 вопроса), ожидаемой/ прогнозируемой (9 вопросов) видов стигмы, а также стереотипов/ предрассудков (6 вопросов) можно определить, взяв среднее значение ответов, полученных соответственно по каждому механизму стигмы. Под-шкалы источников стигмы могут быть созданы для ожидаемой стигмы, взяв среднее значение ответов, полученных на соответствующие вопросы касательно членов семьи (3 пункта), медицинских работников (3 пункта) и других лиц, употребляющих инъекционные наркотики (3 пункта).

**Инструкция:** Метадон – медикамент, который могут применять в лечении опиоидной зависимости. Метадон – синтетический опиоидный агонист, который устраняет симптомы отмены и снимает тягу к потреблению наркотиков, воздействуя на опиоидные рецепторы в головном мозге - те же рецепторы, которые активируют другие опиоиды, такие как героин, морфин и опиоидные обезболивающие. Хотя он прикрепляется к опиоидным рецепторам и активирует их, он делает это медленнее, чем другие опиоиды, и его применение при лечении людей с опиоидной зависимостью в лечебных дозах не вызывает эйфории**. Следующие вопросы я задам Вам о том, как к Вам могли бы относиться, если бы Вы в будущем стали принимать метадон. Пожалуйста, выберите один вариант ответа на каждый вопрос. Не тратьте слишком много времени на обдумывание своего ответа. Ваша первая реакция, зачастую – самый лучший ответ.**

**ОЖИДАЕМАЯ СТРУКТУРНАЯ СТИГМА** (Заголовок можно опустить при администрировании опроса)

|  |  | Совсем не обеспокоен | Немного | В какой-то степени | Достаточно сильно | Чрезвычайно |
| --- | --- | --- | --- | --- | --- | --- |
| 1. | Насколько Вы обеспокоены тем, что милиция будет преследовать Вас, если Вы будете принимать метадон? | 1 | 2 | 3 | 4 | 5 |
| 2. | Насколько Вы обеспокоены тем, что если бы Вы обратитесь за лечением, Вы будете зарегистрированы как человек, принимающий метадон? | 1 | 2 | 3 | 4 | 5 |
| 3. | Насколько Вы обеспокоены тем, что Вам могут откажут в жилье или трудоустройстве в связи с приемом метадона? | 1 | 2 | 3 | 4 | 5 |

**ОЖИДАЕМАЯ МЕЖЛИЧНОСТНАЯ СТИГМА** (Заголовок можно опустить при администрировании опроса)

Насколько велика вероятность того, что люди отнесутся к Вам в будущем следующим образом, если Вы в будущем будете принимать метадон?

|  |  | Очень маловероятно | Маловероятно | Затрудняюсь ответить: ни маловероятно, ни вероятно | Вероятно | Очень вероятно |
| --- | --- | --- | --- | --- | --- | --- |
| 4. | Члены семьи будут считать, что я - все еще потребитель наркотических веществ. | 1 | 2 | 3 | 4 | 5 |
| 5. | Члены семьи не отнесутся с поддержкой к моему лечению метадоном. | 1 | 2 | 3 | 4 | 5 |
| 6. | Члены семьи будут думать, что я не могу выздороветь. | 1 | 2 | 3 | 4 | 5 |
| 7. | Медицинские работники будут считать, что я все еще - потребитель наркотиков. | 1 | 2 | 3 | 4 | 5 |
| 8. | Медицинские работники будут плохо заботиться обо мне. | 1 | 2 | 3 | 4 | 5 |
| 9. | Медицинские работники не будут прописывать нужный мне медикамент. | 1 | 2 | 3 | 4 | 5 |
| 10. | Другие люди, потребляющие наркотические вещества инъекционно, будут считать меня слабым (-ой) (если я буду принимать метадон). | 1 | 2 | 3 | 4 | 5 |
| 11. | Другие люди, потребляющие наркотические вещества, не будут поддерживать меня (если я буду принимать метадон). | 1 | 2 | 3 | 4 | 5 |
| 12. | Другие люди, потребляющие наркотические вещества, не будут доверять мне (если я буду принимать метадон). | 1 | 2 | 3 | 4 | 5 |

**СТЕРЕОТИПЫ И ПРЕДРАССУДКИ** (Заголовок можно опустить при администрировании опроса)

**Инструкции**: Следующие утверждения отражают чувства, которые, как сообщили некоторые люди, они испытывают к людям, принимающим метадон. Пожалуйста, укажите, насколько Вы согласны с каждым утверждением. Не тратьте слишком много времени на обдумывание ответа. Ваше первое впечатление - зачастую самое лучшее.

Как Вы относитесь к людям, которые принимают метадон?

|  |  | Совершено не согласен | Hе согласен | Нейтрален: ни не согласен, ни согласен | Согласен | Полностью согласен |
| --- | --- | --- | --- | --- | --- | --- |
| 13. | Люди, принимающие метадон, замещают один наркотик – другим. | 1 | 2 | 3 | 4 | 5 |
| 14. | Людям, принимающим метадон, нельзя доверять. | 1 | 2 | 3 | 4 | 5 |
| 15. | Люди, принимающие метадон, не контролируют собственную жизнь. | 1 | 2 | 3 | 4 | 5 |
| 16. | Люди, принимающие метадон, вызывают во мне чувство дискомфорта. | 1 | 2 | 3 | 4 | 5 |
| 17. | Люди, принимающие метадон, вселяют в меня надежду. | 1 | 2 | 3 | 4 | 5 |
| 18. | Люди, принимающие метадон, вызывают у меня злость. | 1 | 2 | 3 | 4 | 5 |
