# Supplementary material for: Cohort profile: the Kyrgyzstan InterSectional Stigma (KISS) injection drug use cohort study
Source: Harm Reduct J. 2022 May 25;19:53. doi: 10.1186/s12954-022-00633-5 (PMC9131652; doi:10.1186/s12954-022-00633-5)
Supplement: Supplementary file 3 — Additional file 3. HIV stigma measure: English, Russian [file 12954_2022_633_MOESM3_ESM.docx]

**Additional file 3 (HIV Stigma Measure: English, Russian)**

**Supplementary Material for the Article:**

Cohort Profile: The Kyrgyzstan InterSectional Stigma (KISS) Injection Drug Use Cohort Study

**Author Names and Affiliations:**

Laramie R. Smith^1^, Natalia Shumskaia^2^, Ainura Kurmanalieva^2^, Thomas L. Patterson^3^, Dan Werb^1,4^, Anna Bluym^1^, Angel B. Algarin^1^, Samantha Yeager^1^, and Javier Cepeda^5^

^1^ Division of Infectious Diseases and Global Public Health, University of California, San Diego, La Jolla, CA, USA

^2^ AIDS Foundation – East West in the Kyrgyz Republic, Bishkek, Kyrgyzstan

^3^ Department of Psychiatry, University of California, San Diego, La Jolla, CA, USA

^4^ Centre on Drug Policy Evaluation, St. Michael’s Hospital, Toronto, Canada

^5^ Department of Epidemiology, Johns Hopkins Bloomberg School of Public Health, Baltimore, MD, USA

**Corresponding Author:**

Laramie R Smith, PhD. Associate Professor. Division of Infectious Diseases and Global Public Health, Department of Medicine, University of California San Diego, 9500 Gilman Drive, Mail Code 0507, La Jolla, CA 92093-0507. Phone: +1 858-822-1462. E-mail: [laramie@ucsd.edu](mailto:laramie@ucsd.edu)

**This material supplements, but does not replace, the peer-reviewed paper in the *Harm Reduction Journal* and reflects partial supplemental material previously published in *Addiction*.**

**Multilevel HIV Stigma Mechanism Scale (mHIV-SMS)**

**The original scale:** This scale has been adapted from the original HIV Stigma Mechanisms Scale. The anticipated and internalized stigma items in this measure was developed and originally assessed in the United States (U.S.) among people living with HIV enrolled in HIV care and was published in *AIDS and Behavior,* cited below. The HIV stereotypes and prejudice stigma items in this measure was also developed and originally assessed among substance-using populations enrolled in methadone maintenance treatment in the U.S. and was published in *AIDS Education and Prevention*, cited below. The original scale items were co-developed by Laramie R. Smith, Ph.D. and Valerie A. Earnshaw, Ph.D.

**The current scale:** The Multilevel HIV Stigma Mechanisms Scale (mHIV-SMS) was revised to be implemented with persons who inject drugs regardless of their HIV status. As such, the phrase ‘because of your HIV status’ was replaced with the phrase, ‘if you tested HIV positive and people knew your HIV status’. The current scale was revised by Laramie R. Smith, Ph.D.

The current scale includes the anticipated stigma subscales from two stigma sources (family members, healthcare workers) to assess interpersonal-level manifestations of stigma as measured in the original scale. The current scale includes a third source of anticipated stigma identified in the extant literature as influencing HIV prevention outcomes among PWID (i.e. other persons who inject drugs). This third stigma source replaces ‘community/social workers’ as the third anticipated stigma source subscale assessed in the original version of the scale in the U.S. context.

The current version of the scale also assesses experiences of anticipated structural stigma that were not assessed in the original HIV-SMS. This includes the addition a three-item subscale that measures how concerned the participant is that they will experience structural or system-level consequences if they tested HIV positive and their HIV status was known. These anticipated structural consequences were informed by the extant literature on structural manifestations of stigma in the Eastern European Central Asia context (i.e., police harassment, registration in government-based systems as a person living with HIV, and being denied housing or employment).

Finally, the current scale includes six items to assess individual-level experiences of stigma towards HIV among PWID regardless of their HIV status by assessing the degree to which PWID endorse stereotypes (3-items) and prejudice (3-items) about people living with HIV. In addition, the current scale includes six items to assess internalized HIV stigma among PWID living with HIV.

**Original Scale Citations:**

Earnshaw, V.E, Smith, L.R., Chaudior, S.R., Amico, K.R., & Copenhaver, M.M. (2013). HIV Stigma Mechanisms and Well-being among PLWH: A test of the HIV Stigma Framework*. AIDS and Behavior*, 17(5):1785-1795. PMCID: [PMC3664141](https://www.ncbi.nlm.nih.gov/pubmed/23456594)

Earnshaw, V.A, Smith, L.R., Chaudior, S.R., Lee, I-C, & Copenhaver, M.M. (2012). Stereotypes about PLWH: Implications for perceptions of HIV risk and Testing Frequency. *AIDS* *Education and Prevention,* 24(6):574-581. PMCID**:** [PMC3641644](https://www.ncbi.nlm.nih.gov/pubmed/23206205)

**Intended use:** The mHIV-SMS was developed for use in a diverse range of people with greater vulnerability to HIV transmission, such as people who inject drugs, who are expected to be aware of social stigma towards HIV regardless of their personal HIV status. It may be adapted for persons experienced with other forms of elevated HIV transmission risk accordingly. However, the internalized HIV stigma items should only be assessed among persons living with HIV.

**Scoring:** All responses are given on a 5-point Likert-scale, with higher scores indicating greater endorsement of HIV stigma. Structural (3 items), Anticipated (9 items), Stereotypes/Prejudice (6 items) and Internalized (6 items) scales can be created by taking the average of the item response given for each stigma mechanism respectively. Stigma source sub-scales can be created for Anticipated stigma by taking the average responses given for the family members (3 items), healthcare workers (3 items), and other persons who inject drugs (3 items), item responses respectively.

**English: Multilevel HIV Stigma Mechanism Scale (mHIV-SMS)**

**Instructions:** These questions will ask you how you might be treated in the future if you tested HIV positive and people knew your HIV status. Please select on response option for each question. Do not spend too much time considering your answer. Your first impression is usually best.

**ANTICIPATED STRUCTURAL STIGMA** (header can be omitted in the survey)

|  |  | Not at all | A little bit | Somewhat | Quite a bit | Extremely |
| --- | --- | --- | --- | --- | --- | --- |
| 1. | How concerned are you that the police will harass you if you were HIV positive? | 1 | 2 | 3 | 4 | 5 |
| 2. | How concerned are you that you will be registered as someone living with HIV if you were to seek treatment? | 1 | 2 | 3 | 4 | 5 |
| 3. | How concerned are you that you will be denied housing or employment if you were HIV positive. | 1 | 2 | 3 | 4 | 5 |

**ANTICIPATED INTERPERSONAL STIGMA** (header can be omitted in survey)

How likely is it that people will treat you in the following ways in the future if you tested HIV positive and people knew your HIV status?

|  |  | Very unlikely | Unlikely | Neither unlikely nor likely | Likely | Very Likely |
| --- | --- | --- | --- | --- | --- | --- |
| 4. | Family members will avoid me. | 1 | 2 | 3 | 4 | 5 |
| 5. | Family members will look down on me. | 1 | 2 | 3 | 4 | 5 |
| 6. | Family members will treat me differently. | 1 | 2 | 3 | 4 | 5 |
| 7. | Healthcare workers will not listen to my concerns. | 1 | 2 | 3 | 4 | 5 |
| 8. | Healthcare workers will avoid touching me. | 1 | 2 | 3 | 4 | 5 |
| 9. | Healthcare workers will treat me with less respect. | 1 | 2 | 3 | 4 | 5 |
| 10. | Other people who inject drugs will think I’m dangerous (because I have HIV). | 1 | 2 | 3 | 4 | 5 |
| 11. | Other people who inject drugs will not support me (because I have HIV). | 1 | 2 | 3 | 4 | 5 |
| 12. | Other people who inject drugs won’t trust me (because I have HIV). | 1 | 2 | 3 | 4 | 5 |

**STEREOTYPES AND PREJUDICE** (header can be omitted in survey)

**Instructions:** These next statements reflect the way some people have said they felt about people living with HIV. Please indicate much do you agree with each statement. Do not spend too much time considering your answer. Your first impression is usually best.

How do you **feel** about people who have HIV?

|  |  | Strongly disagree | Disagree | Neither disagree nor agree | Agree | Strongly agree |
| --- | --- | --- | --- | --- | --- | --- |
| 13. | Most people who are HIV positive are gay men. | 1 | 2 | 3 | 4 | 5 |
| 14. | Most people who are HIV positive have slept around a lot. | 1 | 2 | 3 | 4 | 5 |
| 15. | Most people who are HIV positive are prostitutes. | 1 | 2 | 3 | 4 | 5 |
| 16. | People who are HIV positive make me feel uncomfortable. | 1 | 2 | 3 | 4 | 5 |
| 17. | People who are HIV positive make me feel compassionate.****** | 5 | 4 | 3 | 2 | 1 |
| 18. | People who have HIV make me feel afraid. | 1 | 2 | 3 | 4 | 5 |

****NOTE:** The item, “*People who are HIV positive make me feel compassionate*” is reverse coded. This item performed less well in the HIV Stereotypes and Prejudice subscale. Measures of internal reliability (see Table 3: α=0.64, ω=0.66) improved when the item was removed. Staff administering the interview observed some participants struggled switching between positively and negatively framed items. Internal reliability when this item is removed was: α=0.70, ω=0.69. The item was retained in the current analysis.

At the month 3 follow up an additional item from the original HIV stereotypes and prejudice scale was added to the HIV stigma measure: “*People who are HIV positive make me feel nervous”*. This item is not reverse coded and performed well when it replaced the item, “*People who are HIV positive make me feel compassionate*”. The internal reliability when the “nervous’ item replaced the “compassionate” item was: α=0.77, ω=0.74

**INTERNALIZED** (header can be omitted in survey)

**Instructions:** These next statements reflect the way some people have said they felt about their HIV status. Please indicate much do you agree with each statement. Do not spend too much time considering your answer. Your first impression is usually best.

How do you **feel** about having HIV?

|  |  | Strongly disagree | Disagree | Neither disagree nor agree | Agree | Strongly agree |
| --- | --- | --- | --- | --- | --- | --- |
| 19. | Having HIV makes me feel like I’m a bad person. | 1 | 2 | 3 | 4 | 5 |
| 20. | I feel I’m not as good as others because I have HIV. | 1 | 2 | 3 | 4 | 5 |
| 21. | I feel ashamed of having HIV. | 1 | 2 | 3 | 4 | 5 |
| 22. | I think less of myself because I have HIV. | 1 | 2 | 3 | 4 | 5 |
| 23. | Having HIV makes me feel unclean. | 1 | 2 | 3 | 4 | 5 |
| 24. | Having HIV is disgusting to me. | 1 | 2 | 3 | 4 | 5 |

**Russian: Multilevel HIV Stigma Mechanism Scale (mHIV-SMS)**

**Многоуровневая шкала для изучения механизма стигматизации, связанной с ВИЧ (mHIV-SMS)**

**Предназначена для использования:** Шкала mHIV-SMS была разработана для использования среди широкого круга людей с повышенной уязвимостью к передаче ВИЧ, таких как люди, употребляющие инъекционные наркотики, которые, как предполагается, будут осведомлены о социальной стигматизации в отношении ВИЧ, независимо от их личного ВИЧ-статуса. Соответственно, шкала может быть адаптирована для лиц, столкнувшихся с другими формами повышенного риска передачи ВИЧ. Однако элементы интернализованной (внутренней) стигмы (самостигмы), связанной с ВИЧ, следует оценивать только среди лиц, живущих с ВИЧ.

**Оценка:** Все ответы даются по 5-балльной шкале вроде шкалы Лайкерта, где более высокие баллы указывают на большее подтверждение стигматизации, связанной с ВИЧ. Балл для оценки структурной (3 вопроса), ожидаемой/ прогнозируемой (9 вопросов) видов стигмы, стереотипов/ предрассудков (6 вопросов) и интернализованной стигмы (внутренней стигмы/ самостигмы) (6 вопросов) можно определить, взяв среднее значение ответов, полученных соответственно по каждому механизму стигмы. Под-шкалы источников стигмы могут быть созданы для оценки ожидаемой стигмы, взяв среднее значение ответов, данных соответственно на вопросы касательно членов семьи (3 пункта).

**Инструкция:** Следующие вопросы коснутся того, как, на Ваш взгляд, к Вам могли бы отнестись, если предположим в будущем Ваш тест на ВИЧ окажется позитивным и о Вашем статусе узнали бы другие люди. Пожалуйста, выберите один вариант ответа на каждый вопрос. Не задумывайтесь слишком долго над вопросами, первый пришедший на ум ответ, обычно–самый лучший.

**ОЖИДАЕМАЯ СТРУКТУРНАЯ СТИГМА** (Заголовок можно опустить при администрировании опроса)

|  |  | Совсем не обеспокоен | Немного | В какой-то степени | Достаточно сильно | Чрезвычайно |
| --- | --- | --- | --- | --- | --- | --- |
| 1. | Насколько Вы обеспокоены тем, что милиция будет преследовать вас, проявлять жестокость, если Вы окажетесь ВИЧ-позитивным? | 1 | 2 | 3 | 4 | 5 |
| 2. | Насколько Вы обеспокоены тем, что, если бы Вам когда-то пришлось обратитесь за лечением, Вас зарегистрировали бы как человека, живущего с ВИЧ? | 1 | 2 | 3 | 4 | 5 |
| 3. | Насколько Вы обеспокоены тем, что Вам откажут в жилье или трудоустройстве, если Вы были бы ВИЧ-позитивны? | 1 | 2 | 3 | 4 | 5 |

**ОЖИДАЕМАЯ МЕЖЛИЧНОСТНАЯ СТИГМА** (Заголовок можно опустить при администрировании опроса)

Насколько велика вероятность того, что к Вам в будущем могли бы отнестись следующим образом, если бы Ваш тест на ВИЧ оказался позитивным и люди бы знали о Вашем статусе?

|  |  | Очень маловероятно | Маловероятно | Затрудняюсь ответить: ни маловероятно, ни вероятно | Вероятно | Очень вероятно |
| --- | --- | --- | --- | --- | --- | --- |
| 4. | Члены семьи буду избегать меня. | 1 | 2 | 3 | 4 | 5 |
| 5. | Члены семьи буду смотреть на меня свысока. | 1 | 2 | 3 | 4 | 5 |
| 6. | Члены семьи буду относиться ко мне по-другому. | 1 | 2 | 3 | 4 | 5 |
| 7. | Медицинские работники не будут прислушиваться к моим проблемам и опасениям. | 1 | 2 | 3 | 4 | 5 |
| 8. | Медицинские работники будут избегать прикасаться ко мне. | 1 | 2 | 3 | 4 | 5 |
| 9. | Медицинские работники будут относится ко мне с меньшим уважением. | 1 | 2 | 3 | 4 | 5 |
| 10. | Другие люди, употребляющие инъекционные наркотические вещества, будут думать, что я опасен(-а) (потому что у меня ВИЧ). | 1 | 2 | 3 | 4 | 5 |
| 11. | Другие люди, употребляющие инъекционные наркотические вещества, не будут поддерживать меня (потому что у меня ВИЧ). | 1 | 2 | 3 | 4 | 5 |
| 12. | Другие люди, употребляющие инъекционные наркотические вещества, не будут доверять мне (потому что у меня ВИЧ). | 1 | 2 | 3 | 4 | 5 |

**СТЕРЕОТИПЫ И ПРЕДРАССУДКИ** (Заголовок можно опустить при администрировании опроса)

**Инструкции**: Следующие утверждения отражают чувства, которые, как сообщили некоторые люди, они испытывают к людям, живущим с ВИЧ. Пожалуйста, укажите, насколько Вы согласны с каждым утверждением. Не тратьте слишком много времени на обдумывание ответа. Ваше первое впечатление - зачастую самое лучшее.

Как Вы относитесь к людям, живущим с ВИЧ?

|  |  | Совершенно не согласен | Не согласен | Нейтрален: ни не согласен, ни согласен | Согласен | Полностью согласен |
| --- | --- | --- | --- | --- | --- | --- |
| 13. | Большинство людей с ВИЧ-позитивным статусом – гомосексуальные мужчины. | 1 | 2 | 3 | 4 | 5 |
| 14. | Большинство людей с ВИЧ-позитивным статусом имели много беспорядочных половых связей. | 1 | 2 | 3 | 4 | 5 |
| 15. | Большинство людей с ВИЧ-позитивным статусом занимается проституцией. | 1 | 2 | 3 | 4 | 5 |
| 16. | Люди с ВИЧ-позитивным статусом вызывают у меня дискомфорт. | 1 | 2 | 3 | 4 | 5 |
| 17. | Люди с ВИЧ-позитивным статусом вызывают у меня сочувствие и сострадание. ****** | 5 | 4 | 3 | 2 | 1 |
| 18. | Люди с ВИЧ-позитивным статусом вызывают у меня страх. | 1 | 2 | 3 | 4 | 5 |

****ПРИМЕЧАНИЕ**. Утверждение «*Люди, инфицированные ВИЧ, вызывают у меня сочувствие*» кодировано обратным образом (от 5 к 1). Этот вопрос показал худшие результаты в под-шкале «Стереотипы и предубеждения о ВИЧ». Показатели внутренней надежности (см. Таблицу 3: α = 0,64, ω = 0,66) улучшились, когда этот пункт был удален. Сотрудники исследования, проводившие интервью, заметили, что некоторые участники с трудом переключались между позитивно и негативно оформленными элементами. Внутренняя надежность при удалении этого элемента составила: α=0,70, ω=0,69. При проведении данного анализа этот пункт был сохранен.

На 3-м месяце наблюдения к показателю стигмы в связи с ВИЧ был добавлен дополнительный пункт из исходной шкалы стереотипов и предубеждений в отношении ВИЧ: «*Люди, инфицированные ВИЧ, вызывают у меня нервозность/ тревожность*». Этот элемент не кодируется в обратном порядке и показал хорошие результаты, когда этим вопросом заменили ранее используемое утверждение «Люди, инфицированные ВИЧ, вызывают у меня сочувствие». Внутренняя надежность при замене утверждения про «сочувствие» новым утверждением о «нервозности/тревожности» составила: α=0,77, ω=0,74.

**ВНУТРЕННЯЯ СТИГМА (САМОСТИГМА)** (Заголовок можно опустить при администрировании опроса)

**Инструкции**: Следующие утверждения отражают чувства, которые, как сообщили некоторые люди, они испытывают в связи со своим ВИЧ-статусом. Пожалуйста, укажите, насколько Вы согласны с каждым утверждением. Не тратьте слишком много времени на обдумывание ответа. Ваше первое впечатление - зачастую самое лучшее.

Как Вы относитесь к тому, что у вас ВИЧ-положительный статус?

|  |  | Совершенно не согласен | Не согласен | Нейтрален: ни не согласен, ни согласен | Согласен | Полностью согласен |
| --- | --- | --- | --- | --- | --- | --- |
| 19. | Иметь ВИЧ-положительный статус заставляет меня чувствовать себя плохим человеком. | 1 | 2 | 3 | 4 | 5 |
| 20. | Я чувствую, что я хуже других, потому что у меня ВИЧ. | 1 | 2 | 3 | 4 | 5 |
| 21. | Я стыжусь того, что у меня ВИЧ. | 1 | 2 | 3 | 4 | 5 |
| 22. | Я думаю о себе хуже, потому что у меня ВИЧ. | 1 | 2 | 3 | 4 | 5 |
| 23. | Иметь ВИЧ заставляет меня чувствовать себя не чистым, противным. | 1 | 2 | 3 | 4 | 5 |
| 24. | Мне противно иметь ВИЧ. | 1 | 2 | 3 | 4 | 5 |
