# Supplementary material for: Cohort profile: the Kyrgyzstan InterSectional Stigma (KISS) injection drug use cohort study
Source: Harm Reduct J. 2022 May 25;19:53. doi: 10.1186/s12954-022-00633-5 (PMC9131652; doi:10.1186/s12954-022-00633-5)
Supplement: Supplementary file 4 — Additional file 4. Methadone maintenance treatment stigma scale post hoc sensitivity analysis [file 12954_2022_633_MOESM4_ESM.docx]

**Additional file 4 (Methadone Maintenance Treatment Stigma Scale Post-hoc Sensitivity Analysis)**

**Table S1 Baseline Experiences of MMT Stigma Overall and by Urbanicity among PWID Injecting Heroin (*N=*240)**

| **Stigma Type and Mechanism** | **Internal Reliability** | | | | **Total**  ***N=* 240** | **Bishkek**  ***N=124*** | **Chuy Oblast**  ***N=116*** | ***t*_(df)_, *p*-value** |
| --- | --- | --- | --- | --- | --- | --- | --- | --- |
|  | **No. Items** | ***α*** | ***ω*** | ***n*** | ***M*, (*SD*)** | ***M*, (*SD*)** | ***M*, (*SD*)** |  |
| **MMT Stigma** |  | | | |  |  |  |  |
| Total | 18 | 0.80 | 0.81 | 222 | 3.23 (0.55) | 3.24 (0.55) | 3.22 (0.54) | *t_(220)_*= 0.232, *p=*0.408 |
| Structural | 3 | 0.63 | 0.67 | 231 | 3.02 (1.14) | 3.00 (1.17) | 3.03 (1.12) | *t_(229)_*= -0.198, *p=*0.421 |
| Anticipated | 9 | 0.79 | 0.79 | 230 | 3.36 (0.68) | 3.32 (0.65) | 3.34 (0.63) | *t_(228)_*= 0.469, *p=*0.320 |
| *Family* | 3 | 0.68 | 0.69 | 231 | 3.43 (0.86) | 3.48 (0.92) | 3.38 (0.79) | *t_(228)_*= 0.942, *p=*0.174 |
| *Healthcare workers* | 3 | 0.68 | 0.71 | 236 | 3.35 (0.80) | 3.38 (0.79) | 3.32 (0.82) | *t_(231)_*= 0.528, *p=*0.299 |
| *Other PWID* | 3 | 0.75 | 0.75 | 239 | 3.21 (0.92) | 3.17 (0.92) | 3.25 (0.93) | *t_(235)_*= -0.672, *p=*0.251 |
| Stereotypes & Prejudice | 6 | 0.74 | 0.77 | 237 | 3.14 (0.65) | 3.12 (0.65) | 3.15 (0.65) | *t_(234)_*= -0.341 *p=*0.367 |

Mean stigma scores can be interpreted as 1=very low stigma, 2=low to moderate stigma, 3=moderate stigma, 4=moderate to high stigma, 5=very high stigma. Statistically significant differences are in **bold**.
